# Supplementary material for: Metabolically reprogrammed eosinophils impair T cell immunity and cause chronic skin infection
Source: EMBO Mol Med. 2026 Mar 11;18(4):1292–317. doi: 10.1038/s44321-026-00392-x (PMC13083857; doi:10.1038/s44321-026-00392-x)
Supplement: Supplementary file 1 — Appendix [file 44321_2026_392_MOESM1_ESM.pdf]

Appendix

Appendix Table S1……………………………………………………………………….1-3

Appendix Table S1……………………………………………………………………….4-8

Appendix Table S1: Exact p values

Figure	Panel	Additional information	Comparision	Statistical test	Statistical outcome
**1**	D	None	Day 6 vs Day 14	two-tailed Mann-Whitney U test	0.0075
		None	Day 14 vs Day 20	two-tailed Mann-Whitney U test	0.0681
	E	BM Eos	Day 0 vs Day 6	two-tailed Mann-Whitney U test	0.0035
		BM Eos	Day 0 vs Day 14	two-tailed Mann-Whitney U test	0.0003
		BM EoPs	Day 0 vs Day 6	two-tailed Mann-Whitney U test	0.0485
		BM EoPs	Day 0 vs Day 14	two-tailed Mann-Whitney U test	0.0095
		Blood Eos	Day 0 vs Day 14	two-tailed Mann-Whitney U test	0.0003
		Blood Eos	Day 6 vs Day 14	two-tailed Mann-Whitney U test	0.0426
		Blood Eos	Day 0 vs Day 20	two-tailed Mann-Whitney U test	0.0043
	F	BM Eos	Treated vs untreated	two-tailed Mann-Whitney U test	0.0002
		Blood Eos	Treated vs untreated	two-tailed Mann-Whitney U test	0.0002
		Skin Eos	Treated vs untreated	two-tailed Mann-Whitney U test	0.0286
**2**	A	None	WT Day 6 vs KO Day 6	two-tailed Mann-Whitney U test	0.0052
		None	WT Day 14 vs KO Day 14	two-tailed Mann-Whitney U test	0.0064
	C	BM Eos	WT Day 6 vs KO Day 6	two-tailed Mann-Whitney U test	0.0005
		BM Eos	WT Day 14 vs KO Day 14	two-tailed Mann-Whitney U test	0.0022
		BM EoPs	WT Day 6 vs KO Day 6	two-tailed Mann-Whitney U test	0.0781
		BM EoPs	WT Day 14 vs KO Day 14	two-tailed Mann-Whitney U test	0.0111
		Blood Eos	WT Day 6 vs KO Day 6	two-tailed Mann-Whitney U test	0.0006
		Blood Eos	WT Day 14 vs KO Day 14	two-tailed Mann-Whitney U test	0.0023
**3**	A	Day 37	WT vs KO	two-tailed Mann-Whitney U test	0.0017
		Day 41	WT vs KO	two-tailed Mann-Whitney U test	0.0001
		Day 44	WT vs KO	two-tailed Mann-Whitney U test	0.0014
		Day 48	WT vs KO	two-tailed Mann-Whitney U test	<0.0001
		Day 51	WT vs KO	two-tailed Mann-Whitney U test	0.0017
		Day 94	WT vs KO	two-tailed Mann-Whitney U test	0.0056
		Day 101	WT vs KO	two-tailed Mann-Whitney U test	0.0007
		Day 107	WT vs KO	two-tailed Mann-Whitney U test	0.0002
		Day 115	WT vs KO	two-tailed Mann-Whitney U test	0.0008
		Day 121	WT vs KO	two-tailed Mann-Whitney U test	<0.0001
	B	Foot skin	Day 95-125 WT vs KO	two-tailed Mann-Whitney U test	0.0077
		dLN	Day 95-125 WT vs KO	two-tailed Mann-Whitney U test	0.0099
		Spleen	Day 95-125 WT vs KO	two-tailed Mann-Whitney U test	0.0188
	C	Day 6	Treated vs untreated	Multiple unpaired t test	0.0020
		Day 13	Treated vs untreated	Multiple unpaired t test	0.0002
		Day 20	Treated vs untreated	Multiple unpaired t test	0.0038
		Day 27	Treated vs untreated	Multiple unpaired t test	0.0417
		Day 36	Treated vs untreated	Multiple unpaired t test	0.0174
		Day 54	Treated vs untreated	Multiple unpaired t test	0.0023
		Day 60	Treated vs untreated	Multiple unpaired t test	0.0004
		Day 67	Treated vs untreated	Multiple unpaired t test	0.0003

		Day 74	Treated vs untreated	Multiple unpaired t test	0.0009
		Day 81	Treated vs untreated	Multiple unpaired t test	<0.0001
		Day 88	Treated vs untreated	Multiple unpaired t test	0.0089
		Day 94	Treated vs untreated	Multiple unpaired t test	0.0034
		Day 103	Treated vs untreated	Multiple unpaired t test	0.0024
		Day 110	Treated vs untreated	Multiple unpaired t test	0.0005
		Day 115	Treated vs untreated	Multiple unpaired t test	0.0004
		Day 122	Treated vs untreated	Multiple unpaired t test	0.0001
		Day 136	Treated vs untreated	Multiple unpaired t test	<0.0001
		Day 143	Treated vs untreated	Multiple unpaired t test	<0.0001
	D	Foot skin	Day 81-85 treated vs untreated	two-tailed Mann-Whitney U test	0.0006
		Foot skin	Day 145 treated vs untreated	two-tailed Mann-Whitney U test	0.0571
		dLN	Day 81-85 treated vs untreated	two-tailed Mann-Whitney U test	0.0059
		dLN	Day 145 treated vs untreated	two-tailed Mann-Whitney U test	0.0571
		Spleen	Day 81-85 treated vs untreated	two-tailed Mann-Whitney U test	0.0133
		Spleen	Day 145 treated vs untreated	two-tailed Mann-Whitney U test	0.0285
	E	Ifng	Day 7 WT vs KO	two-tailed Mann-Whitney U test	0.0013
		Ifng	Day 14 WT vs KO	two-tailed Mann-Whitney U test	0.0379
		Ifng	Day 29 WT vs KO	two-tailed Mann-Whitney U test	0.0021
		Ifng	Day 48 WT vs KO	two-tailed Mann-Whitney U test	0.0095
		Il4	Day 7 WT vs KO	two-tailed Mann-Whitney U test	0.0001
		Il4	Day 14 WT vs KO	two-tailed Mann-Whitney U test	0.0144
		Il4	Day 29 WT vs KO	two-tailed Mann-Whitney U test	0.1000
		Il4	Day 48 WT vs KO	two-tailed Mann-Whitney U test	0.0021
		Il13	Day 7 WT vs KO	two-tailed Mann-Whitney U test	0.0001
		Il13	Day 14 WT vs KO	two-tailed Mann-Whitney U test	0.0104
		Il13	Day 29 WT vs KO	two-tailed Mann-Whitney U test	0.0021
		Il13	Day 48 WT vs KO	two-tailed Mann-Whitney U test	0.0011
		Il10	Day 7 WT vs KO	two-tailed Mann-Whitney U test	0.0001
		Il10	Day 14 WT vs KO	two-tailed Mann-Whitney U test	0.0069
		Il10	Day 29 WT vs KO	two-tailed Mann-Whitney U test	0.0021
		Il10	Day 48 WT vs KO	two-tailed Mann-Whitney U test	0.0043
**5**	F	None	WT vs KO	two-tailed Mann-Whitney U test	0.0289
**6**	D	None	CD4+ cells	Multiple unpaired t test	0.0249
		None	CD4+ CD44+ cells	Multiple unpaired t test	0.0053
	F	Il2	Day 7 WT vs KO	two-tailed Mann-Whitney U test	0.0001
		Il2	Day 14 WT vs KO	two-tailed Mann-Whitney U test	0.0003
		Il2	Day 76-113 WT vs KO	two-tailed Mann-Whitney U test	0.0086
		Tbx21	Day 7 WT vs KO	two-tailed Mann-Whitney U test	0.0649
		Tbx21	Day 14 WT vs KO	two-tailed Mann-Whitney U test	0.0031

		Tbx21	Day 76-113 WT vs KO	two-tailed Mann-Whitney U test	0.0021
	G	None	WT vs KO	two-tailed Mann-Whitney U test	0.0159
**7**	D	None	Eosinophils vs CD4+ cells	two-tailed Mann-Whitney U test	0.0002
	E	None	Eosinophils vs CD4+ cells	two-tailed Mann-Whitney U test	<0.0001
		None	Eosinophils vs Mono.	two-tailed Mann-Whitney U test	<0.0001
	F	CD4+ 2-NBDG+ cells	WT vs KO	two-tailed Mann-Whitney U test	0.0286
		MFI 2-NBDG CD4+ cells	WT vs KO	two-tailed Mann-Whitney U test	0.0286
	H	IFNg+ cells	With vs without Eos	two-tailed Mann-Whitney U test	0.0019
		MFI CD44+ CD4+	With vs without Eos	two-tailed Mann-Whitney U test	0.0017
	I	IFNg+ cells	1:1 vs 5:1	two-tailed Mann-Whitney U test	0.0079
		MFI CD44+ CD4+	1:1 vs 5:1	two-tailed Mann-Whitney U test	0.0159
	J	MFI CD44+ CD4+	Th1 vs Th2	two-tailed Mann-Whitney U test	0.0022
		MFI CD69+ CD4+	Th1 vs Th2	two-tailed Mann-Whitney U test	0.0022
**EV1**	D	BM Eos	Day 0 vs KO Day 14	two-tailed Mann-Whitney U test	0.0333
			WT Day 14 vs KO Day 14	two-tailed Mann-Whitney U test	0.0012
		Skin Eos	WT Day 6 vs KO Day 6	two-tailed Mann-Whitney U test	0.0571
			WT Day 14 vs KO Day 14	two-tailed Mann-Whitney U test	0.0286
**EV5**	H	None	Eosinophils vs CD4+ cells	two-tailed Mann-Whitney U test	<0.0001
	I	MFI CD44+ CD4+	Th1 with vs without Eos	two-tailed Mann-Whitney U test	0.0238
			Th2 with vs without Eos	two-tailed Mann-Whitney U test	0.0238
		MFI CD69+ CD4+	Th1 with vs without Eos	two-tailed Mann-Whitney U test	0.0238
			Th2 with vs without Eos	two-tailed Mann-Whitney U test	0.0119

Appendix Table S2: Exact n values

**Figure 2C**	BM Eos					
	Days post infection	0	Day 6		Day 14	
		Naive	WT	KO	WT	KO
		4,67	5,08	1,83	8,78	3,93
			6,36	1,73	7,42	4,54
		5,63	8,36	2,9	12,6	5,63
		5,34	6,79	0,93	10,1	3,77
			8,17	5,04	11	9,18
				4,93	16,5	6,87
			8,74	4,52	11,2	8,23
			10,8	5,08		6,85
	BM EoP					
	Days post infection	0	Day 6		Day 14	
		Naive	WT	KO	WT	KO
		19,9		8,22	39	
		22,8	26,3	10,8	45	29,6
		18,5	32,5	10,6	52,9	32,1
		12,6	21,9		37,7	22,6
			34,9	33,7	42,6	43
			27,7	31,1	52,3	38
			40,3	27,7	38,4	33,9
			43,8	25,2		35,2
	Blood					
	Days post infection	0	Day 6		Day 14	
		Naive	WT	KO	WT	KO
		1,73	2,03	0,41	3,7	1,51
		1,73	3,21	1,96	2,29	1,73
				1,41	5,99	3,01
			3,54	1,82	3,61	0,77
			5,6	2,25	7,58	1,77
			3,81	1,76	6,14	1,89
			6,99	1,39		
			4,29	1,36	8,38	2,52

Figure 3E	*Ifng*								
	Days post infection	B/6 4get							
	0	3,97	1,97	0					
	7	0	0	1,09	0,28	0,26	0		
	14	0	6,91	40,02	16,98	27,81	26,29	17,94	3,08
	29	4,35	2,55	14,91	0,60	2,17	3,38		
	48		1,64	2,86	3,2799306	3,99898292			
	76-113	3605,64	1964,58	4718,36	2709,32	3032,01			
		B/6 dbl-Gata-4get							
	0	0	8,36	5,12					
	7	28,36	0,34	2,70	5,36	350,33	169,08	120,35	14,16
	14	569,41	2,79	189,36	1376,21	796,00	15,08	717,70	617,92
	29	1303,90	321,94	3681,04	834,73	2564,92	1236,28		
	48	2837,58	983,05	1389,61	3043,49	3259,47	3525,46		
	76-113	3763,17	1732,19	1184,79	3928,74	3880,86	3493,02		
Il4									
	Days post infection	B/6 4get							
	0	0	0	2,61					
	7	0,55	0	0,711	0,69	0,60	0,55	0,47	0
	14	0	1,22	0	1,49	4,15	4,43	8,02	6,07
	29	1,47	0	0					
	48	11,19	1,55	3,64	19,66	54,53	6,09		
	76-113	90,36	85,59	116,42	85,49	56,90	211,51		
		B/6 dbl-Gata-4get							
	0	3,31	0	0					
	7	7,45	3,84	8,82	13,08	28,49	32,55	36,72	13,29
	14	70,24	3,70	4,19	64,48	92,95	5,94	98,14	70,58
	29	122,16	62,31	154,93					
	48	65,79	283,75	224,66	315,19	417,76	189,74		
	76-113	310,95	134,45	117,30	165,71	116,78	153,52		
Il13									
	Days post infection	B/6 4get							
	0	33,35	16,06	18,34	16,27				
	7	17,64	0	16,98	21,70	14,67	44,54	37,98	35,34
	14	36,30	290,71	0	33,31	214,00	131,92	138,97	82,45
	29	209,27	36,75	88,43	230,42	35,35	298,02		
	48	227,31	157,61	134,07	217,48	514,45	142,15	129,86	
	76-113	301,12	196,61	818,93	154,78	354,55			
		B/6 dbl-Gata-4get							

	0	35,94	23,79	18,05	16,27	9,4			
	7	142,89	48,82	269,31	231,89	199,32	630,68	418,70	117,28
	14	2836,74	249,87	143,82	2408,38	3712,14	75,05	2238,39	434,77
	29	4373,14	2406,91	518,85	1559,40	2219,66	2032,23		
	48	3931,05	3717,63	772,33	3902,98	1725,58	2282,32		
	76-113	779,14	150,14	619,59	1336,01	223,24	182,82		
Il10									
	Days post infection	B/6 4get							
	0	5,23	6,42	6,58					
	7	0	0	0,79	0,67	0,22	0	0	0
	14	0	6,91	16,98	4,78	7,16	0,90	2,64	
	29	2,51	2,61	1,52	4,31	5,48	12,27		
	48	1,05	7,56	7,89	3,06	12,60			
	76-113	186,04	138,15	132,72	109,74	88,37	163,12		
		B/6 dbl-Gata-4get							
	0	29,37	17,91						
	7	17,94	2,39	2,64	9,20	77,56	63,22	34,75	7,35
	14	569,41	2,79	189,36	19,49	20,12	53,38	66,81	
	29	41,28	16,90	56,63	249,21	340,30	171,68		
	48	112,18	312,61	183,50	251,28	320,54	188,56		
	76-113	563,47	287,59	469,03	254,07	262,08	171,57		

**Figure 5H**	***Arg1***								
	Days post infection	B/6 4get							
	0	12001	9497	6833					
	7	8416	17011	20866	11344	13085	15054	18001	20645
	14	38501	30493	71535	20256	38563	54378	64983	71971
	29	157530	25204	63885	61949	44572	70294		
	48	141953	28031	50374	133021	199803	79845		
	76-113	376083	330644	696275	165188	202256	410620		
		B/6 dbl-Gata-4get							
	0	13896	14599						
	7	7922	7822	11387	6645	11302	14544	13727	9746
	14	65458	10724	6721	45016	12334	107518	22752	
	29	150397	167956	254764	15713	223432	141457		
	48	293877	146293	460084	422030	115987			
	76-113	498677	54067	63609	225473	152054	132927		
	Nos2								
	Days post infection	B/6 4get							

	0	87,32	85,24	58,07					
	7	21,72	36,57	70,98	54,03	19,24	122,76	30,84	30,21
	14	11,00	9,98	37,97	12,50	34,04	24,83	26,38	
	29	17,77	31,03	36,87	24,43	19,23			
	48	39,05	68,18	32,22	47,11				
	76-113	44247	25373	41971	31487	63728			
		B/6 dbl-Gata-4get							
	0	61,434	57,50						
	7	173,24	56,54	126,68	71,59	558,93	266,59	450,48	52,58
	14	1557	69,11	229,29	908,09	65,06	3518	1563	
	29	3846	735	17511	8108	23387	38878		
	48	43809	7923	21356	84967	80995	58408		
	76-113	132093	35467	16285	87832	60188	71377		

**Figure 6F**	***Il2***								
	Days post infection	B/6 4get							
	0	4,04	5,78	3,71					
	7	0	0	1,01	0,60	1,21	0,75	0,64	0,00
	14		4,94	0	9,57	5,02	4,32	3,22	5,25
	29				6,68	5,00	1,14		
	48				2,91	5,89	1,26		
	76-113	4,59	17,80	5,22	38,50	56,24	7,23		
		B/6 dbl-Gata-4get							
	0	4,04	5,78	3,71					
	7	5,20	2,34	5,24	6,25	22,49	12,31	9,71	9,76
	14	111,19	13,53	44,64	70,34	29,20	15,39	31,13	29,30
	29				36,89	80,61	82,90		
	48				38,59	44,29	30,24		
	76-113	53,07	54,80	61,62	62,79	155,66	69,91		
	Tbx21								
	Days post infection	B/6 4get							
	0	64,99	75,92	99,04					
	7	27,81	38,89	88,31	72,01	38,97	19,77	63,70	38,26
	14	8,97	23,24	49,15	28,55	32,47	31,83	32,29	31,69
	29				66,05	20,81	27,08		
	48				26,80		43,01		
	76-113	81,92	133,66	161,21	241,85	210,65	118,46		
		B/6 dbl-Gata-4get							
	0	64,99	75,92	99,04					
	7	139,52	35,65	53,19	64,99	267,17	96,78	126,41	61,15

	14	248,82	34,70	59,78	132,70	319,13	73,42	192,85	195,67
	29				655,60	878,72	621,46		
	48				742,48	809,29	587,70		
	76-113	366,09	383,47	543,83	341,30	770,41	450,68		
