# Supplementary material for: Metabolically reprogrammed eosinophils impair T cell immunity and cause chronic skin infection
Source: EMBO Mol Med. 2026 Mar 11;18(4):1292–317. doi: 10.1038/s44321-026-00392-x (PMC13083857; doi:10.1038/s44321-026-00392-x)
Supplement: Supplementary file 10 — Expanded View Figures [file 44321_2026_392_MOESM10_ESM.pdf]

Expanded View Figures

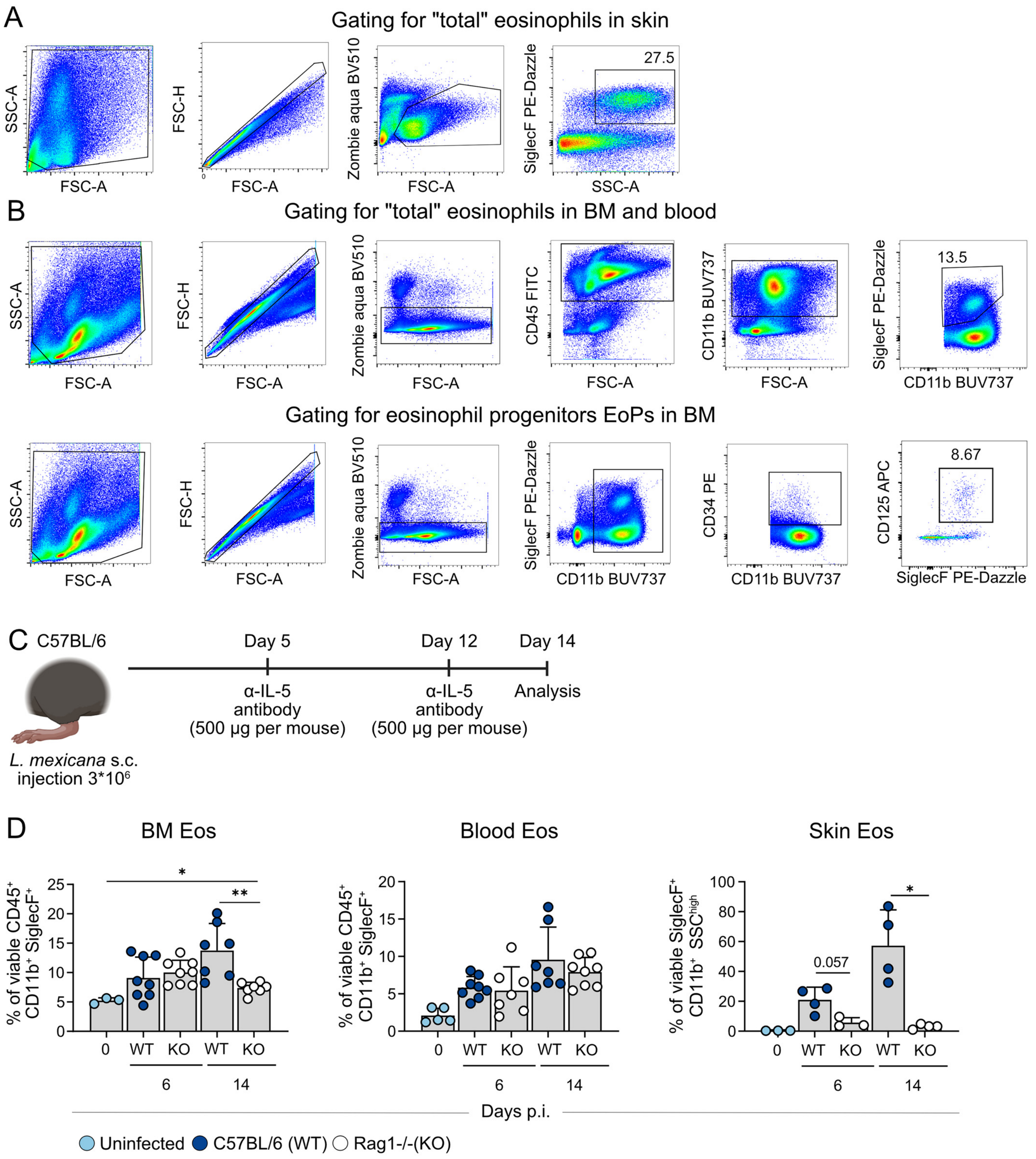

 © The Author(s)

◀ **Figure EV1. Flow cytometric characterization and regulation of eosinophils during *L. mexicana* infection.**

Representative gating strategy for skin (**A**) and BM eosinophil (**B**), as well as eosinophil progenitors (**C**, EoP) at day 14 p.i. **C** C57BL/6 mice were intraperitoneally treated with 500 µg of either isotype control or anti-IL-5 antibody on days 5 and 12 p.i. Flow cytometric analysis was performed on day 14 p.i. with bone marrow, blood and skin lesions cells. (**D**) Flow cytometric analysis of cells from bone marrow, blood and skin lesions of C57BL/6 (WT) and Rag1$^{-/-}$ (KO) mice infected with *L. mexicana* (left panel: day 0 $n = 3$; day 6 WT $n = 8$, KO $n = 8$; day 14 WT $n = 7$, KO $n = 7$; middle panel: day 0 $n = 5$; day 6 WT $n = 8$, KO $n = 7$; day 14 WT $n = 7$, KO $n = 8$; right panel: day 0 $n = 3$; day 6 WT $n = 4$, KO $n = 3$; day 14 WT $n = 4$, 1-2 independent experiments). Data are mean ± s.d. Information regarding the exact p values and the statistical tests performed is provided in the Appendix Table S1.

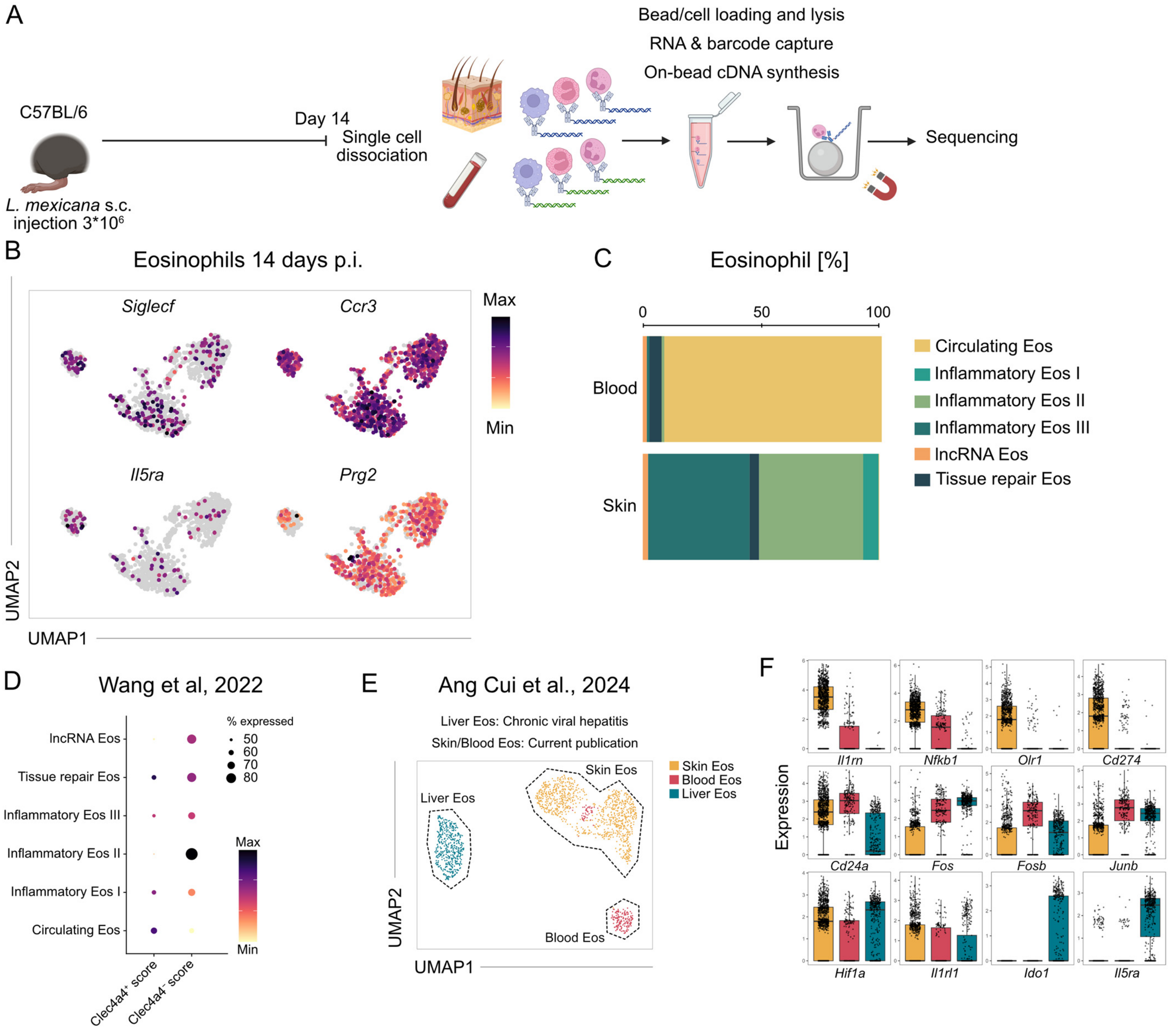

Figure EV2. Experimental and computational workflow for identification and integration of eosinophils from *L. mexicana*-infected skin and blood.

(**A**) Experimental workflow of nanowell-based scRNA Seq of total viable foot skin cells and Percoll-enriched granulocytes of the blood on day 14 p.i. (**B**) Feature plots of canonical eosinophil markers. Expression intensity is represented by a color gradient, with gray indicating no detectable expression. (**C**) Relative abundance of eosinophil subsets across blood and skin during infection, as assessed by scRNA-seq. (**D**) Expression dot plot depicting gene signatures of Clec4a4^{+} and Clec4a4^{-} eosinophil subsets based on published bulk RNA sequencing data from intestinal eosinophils of naive mice (Wang et al, 2022). (**E**) UMAP visualization of the computational integration of the eosinophil subsets from day 14 after *L. mexicana* infection (**B**) with liver eosinophils of hepatitis C patients (Cui et al, 2024). (**F**) Boxplot with jitters representation of differentially expressed genes among eosinophils from the computational integration depicted in (**E**). Box plots show the median (center line) and the interquartile range (box bounds, 25th–75th percentiles). Whiskers extend to the minimum and maximum values within 1.5×IQR of the lower and upper quartiles (Tukey definition). Jittered points represent individual cells ($n = 997$ skin cells, $n = 213$ blood cells, $n = 437$ liver cells).

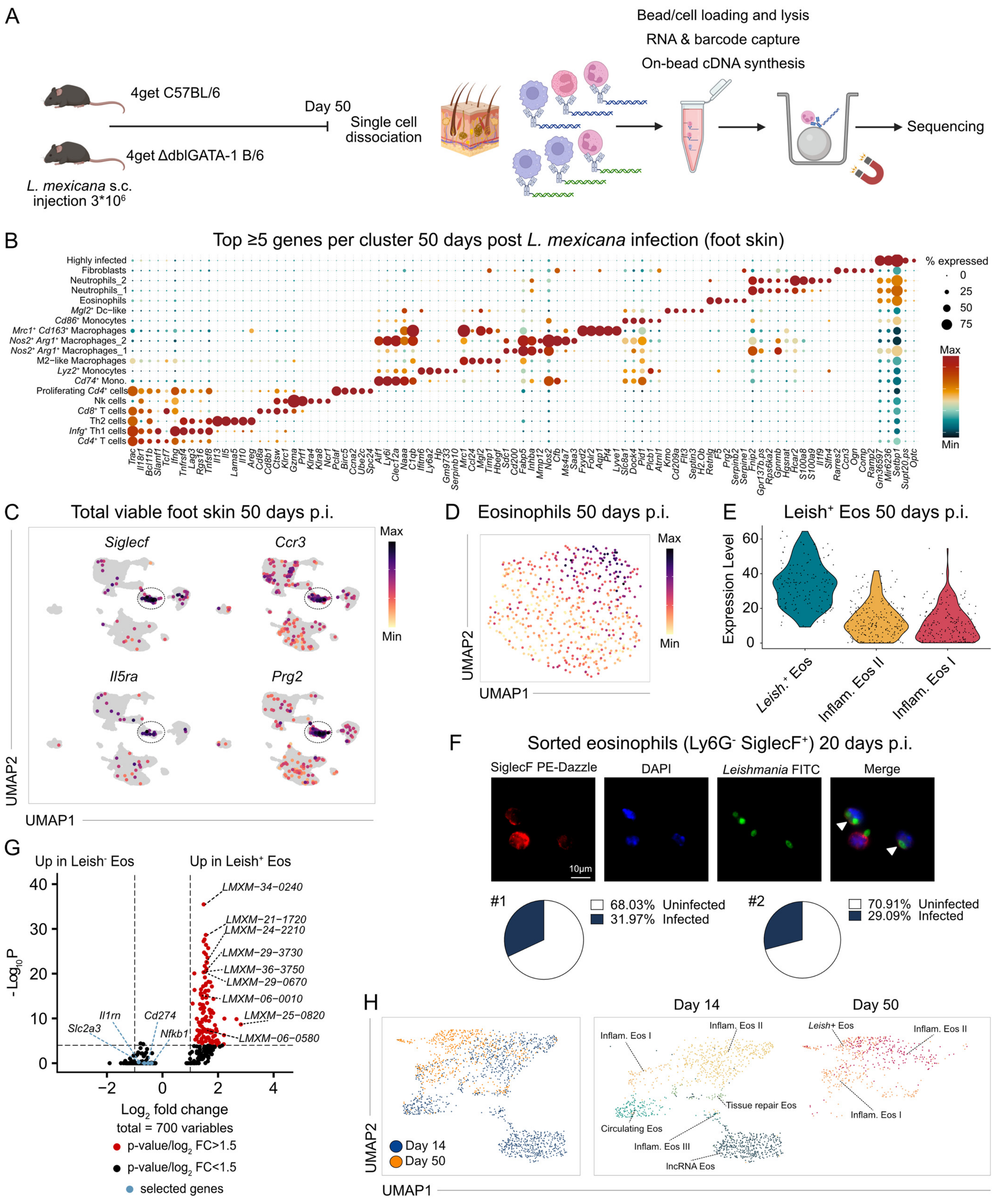
A
4get C57BL/6
4get ΔdblGATA-1 B/6
L. mexicana s.c. injection 3*10^6
Day 50
Single cell dissociation
Bead/cell loading and lysis
RNA & barcode capture
On-bead cDNA synthesis
Sequencing
B
Top ≥5 genes per cluster 50 days post L. mexicana infection (foot skin)
% expressed
Max
Min
C
Total viable foot skin 50 days p.i.
Siglecf
Ccr3
Il5ra
Prg2
UMAP1
UMAP2
D
Eosinophils 50 days p.i.
E
Leish+ Eos 50 days p.i.
Expression Level
Leish.+ Eos
Inflam. Eos II
Inflam. Eos I
F
Sorted eosinophils (Ly6G- SiglecF+) 20 days p.i.
SiglecF PE-Dazzle
DAPI
Leishmania FITC
Merge
10μm
#1
68.03% Uninfected
31.97% Infected
#2
70.91% Uninfected
29.09% Infected
G
Up in Leish- Eos
Up in Leish+ Eos
LMXM−34−0240
LMXM−21−1720
LMXM−24−2210
LMXM−29−3730
LMXM−36−3750
LMXM−29−0670
LMXM−06−0010
LMXM−25−0820
LMXM−06−0580
Il1rn
Cd274
Slc2a3
Nfkb1
-Log10 P
Log2 fold change
total = 700 variables
p-value/log2 FC>1.5
p-value/log2 FC<1.5
selected genes
H
Day 14
Day 50
Inflam. Eos I
Inflam. Eos II
Tissue repair Eos
Circulating Eos
Inflam. Eos III
lncRNA Eos
Leish+ Eos

 © The Author(s)

Figure EV3. Experimental and computational workflow for identification of *L. mexicana*-infected eosinophils at day 50 post infection.

(**A**) Experimental workflow of nanowell-based scRNA Seq of total viable foot skin cells from 4get C57BL/6 and 4get ΔdblGATA-1 B/6 mice on day 50 p.i. (**B**) Expression dot plot depicting the top ≥5 differentially expressed genes for each cluster on day 50 p.i. (total viable foot skin cells). (**C**) Feature plots of canonical eosinophil markers. Expression intensity is represented by a color gradient, with gray indicating no detectable expression. The encircled areas represent the eosinophils. (**D**) Feature plot of all *L. mexicana* transcripts (identified via the prefix *LMXM*) within the eosinophil subset at day 50 p.i. Expression intensity is represented by a color gradient, with gray indicating no detectable expression. (**E**) Ranked violin plot of *L. mexicana* gene expression (prefix *LMXM*) across all identified eosinophil clusters. (**F**) Top: representative images of sorted eosinophils (Ly6G⁻ SiglecF⁺; 20 days p.i.) stained with anti-SiglecF, anti-*Leishmania* and DAPI. Scale bar, 10 µm. Bottom: quantification of the relative abundance of infected eosinophils ($n = 2$ independent experiments, 5 mice pooled each, 200 cells counted per quantification). (**G**) Volcano plot of differentially expressed genes comparing *Leish*⁺ versus non-infected eosinophils. Differential gene expression ($n = 700$ genes) was calculated using Seurat *FindMarkers* with a Wilcoxon rank-sum test; adjusted *p*-values are shown. (**H**) UMAP visualization illustrates the in silico integration of eosinophil transcriptomes from day 14 p.i. (see Fig. 4A) and day 50 p.i. (see Fig. 4E).

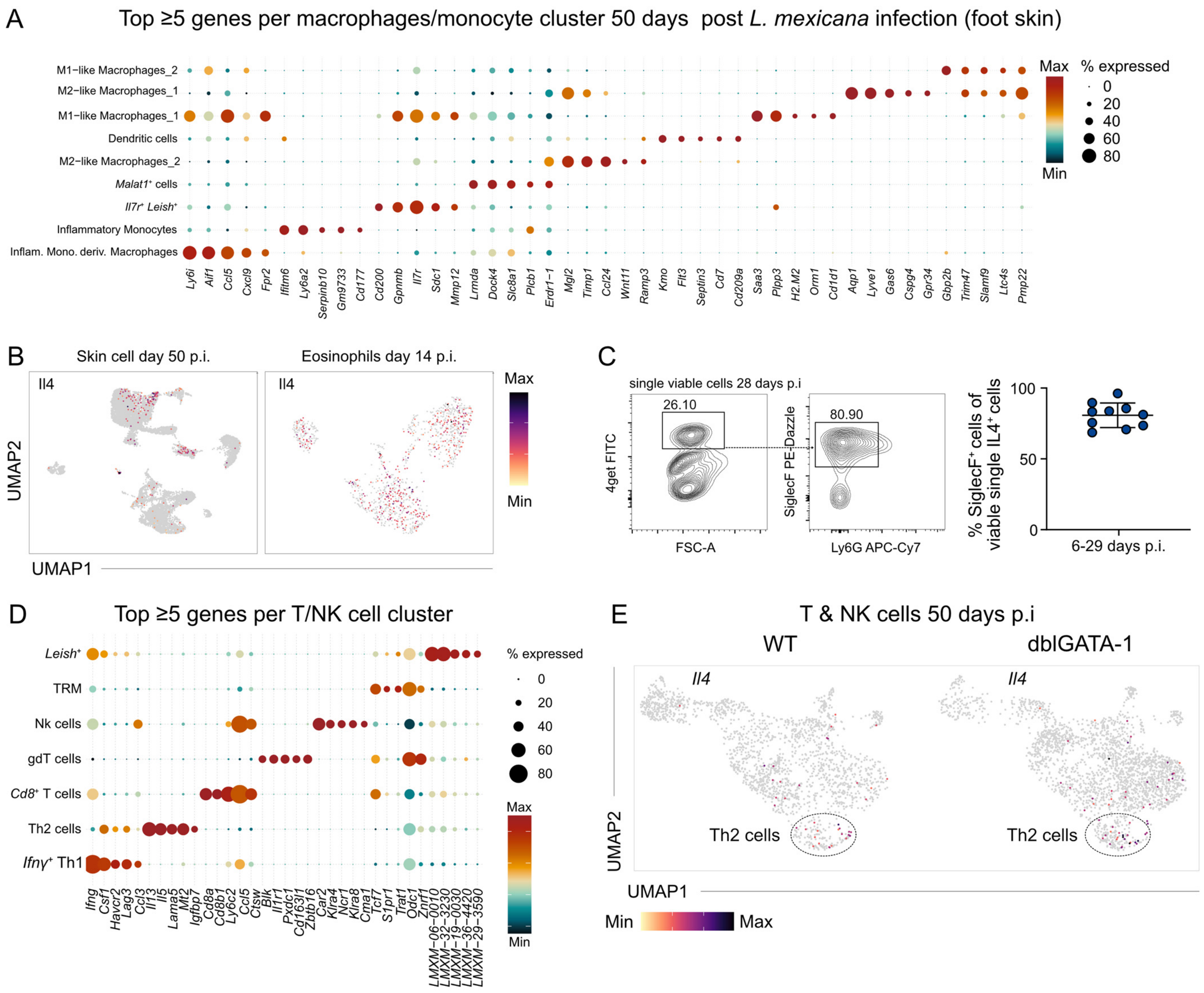

Figure EV4. Transcriptional characterization of immune cell subsets in wild-type and eosinophil-deficient mice at day 50 post infection.

(**A**) Expression dot plot depicting the top ≥5 differentially expressed genes for each myeloid cell cluster on day 50 p.i. (total viable foot skin). (**B**) Feature plots displaying the *Il4* expression overlaid on the UMAP on day 50 p.i. (left) and day 14 p.i. (right). Expression intensity is represented by a color gradient, with gray indicating no detectable expression. (**C**) Left, representative flow cytometric analysis of 4get⁺ (IL-4) SiglecF⁺ cells at day 28 p.i. Right, relative abundance of SiglecF⁺ cells within the 4get⁺ (IL-4⁺) population of total viable foot skin cells at days 6–29 p.i. ($n = 10$, 4 independent experiments, mean ± s.d.). (**D**) Expression dot plot depicting the top ≥5 differentially expressed genes for each T- and NK-cell cluster on day 50 p.i. (total viable foot skin cells). (**E**) Feature plots displaying the expression of *Il4* overlaid on the UMAP of the T- and NK-cell cluster. Expression intensity is represented by a color gradient, with gray indicating no detectable expression.

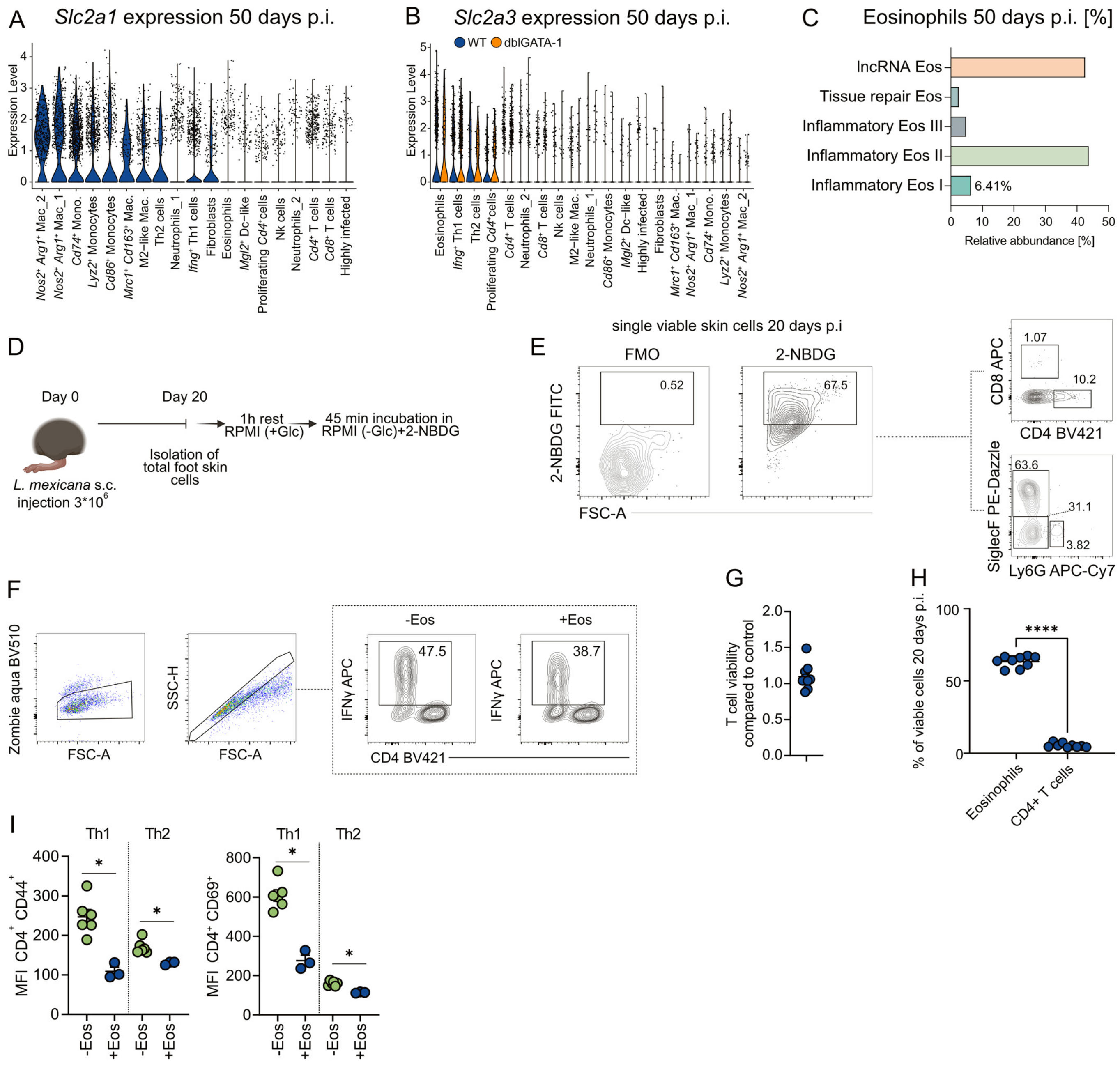

Figure EV5. Identification of a GLUT3⁺ eosinophil subset and its functional impact on Th1 versus Th2 cells during *L. mexicana* infection.

(**A**) Ranked violin plot of the *Slc2a1* (solute carrier family 2 member 1, glucose transporter 1) expression within all identified clusters at day 50 p.i. (total viable foot skin cells). (**B**) Ranked violin plot of the *Slc2a3* (solute carrier family 2 member 3, glucose transporter 3) expression within all identified clusters at day 50 p.i. (total viable foot skin cells) comparing WT vs dblGATA-1 mice. (**C**) Relative abundance of eosinophil subsets in the skin during infection, as assessed by scRNA-seq. (**D**) Experimental workflow of the 2-NBDG assay (Glc, glucose). (**E**) Representative flow cytometric analysis of 2-NBDG uptake by ex vivo cultured cells from day 20 p.i. (**F**) Exemplary gating strategy for the eosinophils/Th1 co-culture. (**G**) Flow cytometric quantification of the T cell viability in the eosinophil/T cell co-culture assay in a 1:5 ratio ($n = 9$, 2 independent experiments). (**H**) Flow cytometric quantification of the relative abundance of CD11b⁺ SiglecF⁺ eosinophils and CD3⁺ CD4⁺ T cells 20 days p. i. in *L. mexicana*-induced skin lesions ($n = 9$, 2 independent experiments). (**I**) Flow cytometric quantification of CD44 (left) and CD69 (right) MFI on CD4⁺ T cells in the eosinophil/T-cell co-culture assay using either generated Th1 cells or Th2 cells at a 1:5 ratio (-Eos: $n = 6$; +Eos: $n = 3$, 1 of 2 representative experiments). Data are mean ± s.d. (error bars fall within the symbols). Information regarding the exact p values and the statistical tests performed is provided in the Appendix Table S1.

 © The Author(s)
